# Response to Elwood, M. et al., Comment on: Maternal Exposure to Domestic Hair Cosmetics and Occupational Endocrine Disruptors Is Associated with a Higher Risk of Hypospadias in the Offspring. *Int. J. Environ. Res. Public Health* 2017, *14*, 27

**DOI:** 10.3390/ijerph14091071

**Published:** 2017-09-15

**Authors:** Elodie Haraux, Karine Braun, Philippe Buisson, Erwan Stéphan-Blanchard, Camille Devauchelle, Jannick Ricard, Bernard Boudailliez, Pierre Tourneux, Richard Gouron, Karen Chardon

**Affiliations:** 1 Department of Paediatric Surgery, Amiens University Hospital, 80054 Amiens, France; buisson.philippe@chu-amiens.fr (P.B.); ricard.jannick@chu-amiens.fr (J.R.); gouron.richard@chu-amiens.fr (R.G.); 2 Department of Paediatrics, Amiens University Hospital, 80054 Amiens, France; braun.karine@chu-amiens.fr (K.B.); boudailliez.bernard@chu-amiens.fr (B.B.); 3 PériTox-INERIS Laboratory, Jules Verne University of Picardy, 80054 Amiens, France; erwan.stephan@u-picardie.fr (E.S.-B.); karen.chardon@u-picardie.fr (K.C.); 4 Department of Paediatrics, Creil Hospital, 60100 Creil, France; camille.devauchelle@ch-creil.fr; 5 Department of Paediatric Intensive Care Unit, Amiens University Hospital, 80054 Amiens, France; tourneux.pierre@chu-amiens.fr

Dear Editor,

Thank you for inviting us to reply to a “Comment” paper to our published paper “Maternal Exposure to Domestic Hair Cosmetics and Occupational Endocrine Disruptors Is Associated with a Higher Risk of Hypospadias in the Offspring” (Authors: Elodie Haraux, Karine Braun, Philippe Buisson, Erwan Stéphan-Blanchard, Jannick Ricard, Camille Devauchelle, Bernard Boudailliez, Pierre Tourneux, Richard Gouron, Karen Chardon).

We thank the comment’s authors for highlighting some inconsistencies in the published results [1]. After verification, we acknowledge that incorrect data were reported in Table 4, and could have led to misinterpretation of our results. A corrected version of this table is provided below. Importantly, these changes do not modify the significance of the results or their related conclusions. Also, there is now no significant difference for missing data on use of hair cosmetics between cases (17.5%) and controls (18.5%). Our study is the first to report an impact of the use of domestic hair cosmetics (not only EDCs occupational exposure *per se*) on the risk of hypospadias.

As pointed out by the comment’s authors, the participation rate was not recorded in our study but this limitation was clearly indicated in the ‘Discussion’ section of the manuscript.

As questioned by the comment’s authors, the strong increase between the univariate and multivariate OR for EDCs occupational exposure was mainly due to paternal weight. This specific point was discussed in the ‘Parental risk factors and medication’ section of the discussion.

Concerning occupational maternal exposure, we first calculated only for women who worked, and secondly for all women. So when women were housewives, we considered no occupational exposure. These results were presented in Table 4. We also acknowledge that incorrect data were reported about “working during pregnancy” (23 instead of 19 women were housewives in the Hypospadias group vs. 76 and not 65 in the control group). OR was higher when calculated for “working women” (univariate 3.6 (1.4–9.3) and multivariate 9.6 (1.41–66.09)) than for all the women (univariate 3.1 (1.3–7.6) and 5.1 (1.1–23.06)). It was a choice to present the first result but in both cases, the association between occupational exposure and hypospadias persisted.

The authors would like to apologize for any inconvenience caused to the readers.

Elodie Haraux and co-authors.

## Figures and Tables

**Table 4:** Univariate analysis of the association between pollutant exposures during the first trimester of pregnancy and the incidence of hypospadias.

		Cases (*n* = 57)	Controls (*n* = 162)	*p*-Value, OR (95% CI)
**COSMETICS**				
**Hair cosmetics (*n*)**	Yes	25	51	0.07
	No	22	81	1.9 (0.9–3.5)
**-Hairspray (*n*)**	Yes	16	37	0.4
	No	31	95	1.3 (0.6–2.7)
**-Colouring shampoo (*n*)**	Yes	13	25	0.2
	No	34	105	1.6 (0.7–3.5)
**CHEMICALS**				
**Chemicals (*n*)**	Yes	41	120	0.38
	No	6	11	0.6 (0.2–1.8)
**-Paint/solvents/gasoline (*n*)**	Yes	5	23	0.3
	No	42	108	0.6 (0.2–1.6)
**-Ink (*n*)**	Yes	3	6	0.6
	No	44	124	NA
**-Glue (*n*)**	Yes	5	13	0.90
	No	42	118	1.1 (0.4–3.2)
**-Household products (*n*)**	Yes	41	120	0.6
	No	5	11	0.8 (0.2–2.3)
**Human antiparasitic (*n*)**	Yes	13	57	0.10
	No	38	93	0.56 (0.3–1.1)
**ENVIRONMENTAL FACTORS**				
**Living <1 km from a field**	Yes	33	76	0.07
	No	14	61	1.9 (0.9–3.8)
**Living <1 km from a factory**	Yes	12	43	0.3
	No	31	78	0.7 (0.3–1.5)
**Garden (*n*)**	Yes	40	99	0.04 *
	No	8	46	2.3 (1.0–5.3)
**Pets (*n*)**	Yes	38	85	0.02 *
	No	13	65	2.2 (1.1–4.5)
**Veterinary insecticides (*n*)**	Yes	37	84	0.04 *
	No	14	65	2.0 (1.02–4.1)
**OCCUPATIONAL FACTORS**				
**Working during pregnancy (*n*)**	Yes	32	86	0.6
	No	23	76	1.2 (0.7–2.3)
**Occupational exposure to EDCs (JEM) (*n*)**	Yes	11	12	0.007 **
	No	44	150	3.6 (1.4–9.3)

Reference: * *p* < 0.05; ** *p* < 0.01.
